# Use of traditional complementary and alternative medicine for HIV patients in KwaZulu-Natal, South Africa

**DOI:** 10.1186/1471-2458-8-255

**Published:** 2008-07-24

**Authors:** Karl Peltzer, Natalie Friend-du Preez, Shandir Ramlagan, Henry Fomundam

**Affiliations:** 1 Health Systems Research Unit, Social Aspect of HIV/AIDS and Health, Human Sciences Research Council, Pretoria, South Africa; 2 Department of Psychology, University of the Free State, Bloemfontein, South Africa; 3 Centre for the Study of Sexual Health & HIV, Homerton University Hospital NHS Foundation Trust, London, UK; 4 HIV/AIDS Pharmaceutical Care Program, Howard University, Pretoria, South Africa

## Abstract

**Background:**

Traditional medicine use has been reported is common among individuals with moderate and advanced HIV disease. The aim of this cross-sectional study was to assess the use of Traditional Complementary and Alternative Medicine (TCAM) for HIV patients prior to initiating antiretroviral therapy in three public hospitals in KwaZulu-Natal, South Africa.

**Methods:**

Using systematic sampling, 618 HIV-positive patients were selected from outpatient departments from three hospitals and interviewed with a questionnaire.

**Results:**

TCAM was commonly used for HIV in the past six months by study participants (317, 51.3%) and herbal therapies alone (183, 29.6%). The use of micronutrients (42.9%) was excluded from TCAM since mostly vitamins were provided by the health facility. Herbal therapies were the most expensive, costing on average 128 Rand (US$16) per patient per month. Most participants (90%) indicated that their health care provider was not aware that they were taking herbal therapies for HIV (90%). Herbal therapies were mainly used for pain relief (87.1%) and spiritual practices or prayer for stress relief (77.6%). Multivariate logistic regression with use of herbs for HIV as the dependent variable identified being on a disability grant and fewer clinic visits to be associated with use of herbs, and TCAM use for HIV identified being on a disability grant, number of HIV symptoms and family members not contributing to main source of household income to be associated with TCAM use.

**Conclusion:**

Traditional herbal therapies and TCAM are commonly used by HIV treatment naïve outpatients of public health facilities in South Africa. Health care providers should routinely screen patients on TCAM use when initiating ART and also during follow-up and monitoring keeping in mind that these patients may not fully disclose other therapies.

## Background

In November 2003, the South African Cabinet approved The Operational Plan for Comprehensive HIV and AIDS Care, Management and Treatment for South Africa [1]. In this plan the proposed range of care for patients encompassed a broad range of treatment options. South Africans living with HIV/AIDS are now encouraged to make their own informed choices about the types of treatment they wish to seek including antiretrovirals (ART), exercise, nutrition, as well as traditional and complementary medicines (TCAM).

Towards the end of the 1990s, the total number of traditional healers in South Africa was estimated to be around 350,000 [2] and an estimated 70 to 80% of South Africans consult traditional healers [3,4]. The freedom of choice for patients to select their treatment modality, the availability and easy access to traditional medicine, the cultural beliefs and preponderance of TCAM use increase the likelihood that ARTs are used with other medicines in South Africa. Studies outside of South Africa have found high rates of TCAM utilization amongst people living with HIV ranging from 15% to 79%, with some researchers suggesting that people infected with HIV use TCAM at substantially higher rates than people with other serious illnesses [5-7].

Anecdotal evidence from South Africa suggests that a number of ART patients resort to traditional medicine after experiencing side effects from ART. Among such patients, few data are available on the prevalence and patterns of traditional and alternative therapy use. The South African National ARV Treatment Guidelines [8] state that patients must disclose any over-the-counter drugs and traditional medicines due to the possibility of contraindications and adverse drug interactions. However, previously published research in the United States has found that up to 70% of patients who use such therapies do not tell their doctors [9]. Potential harmful interactions between some TCAM and ARVs have been shown for garlic, St. John's Wort [10], *Hypoxis *(African potato) and *Sutherlandia frutescens *[11], as well as vitamins and cannabis [5]. It is also important to note that many of the herbal medicines used by several people have not been quantified nor analyzed for its active and inactive components. Langlois-Klassen, Kipp, Jhangri and Rubaale [12] studied the use of traditional herbal medicine by 137 AIDS patients in Kabarole District, western Uganda. Overall, 63.5% of AIDS patients had used herbal medicine after HIV diagnosis for any ailment. Babb et al. [13] studied individuals (n = 44) with moderate or advanced HIV disease attending a workplace clinic providing ART in South Africa, and found that 32% were using traditional medicines, most frequently African potato (9/14) and Aloe vera (3/14), and Malangu [14] found among HIV-infected patients on ART (n = 180) in Pretoria, South Africa, that 4.4% used African traditional medicine and 3.3% complementary and alternative medicines. Chopra [15] found that awareness of antiretroviral therapy in South Africa was generally poor. Antiretroviral drugs were not perceived as new, but one of many alternative therapies for HIV and AIDS. Respondents had more detailed knowledge of indications, effects and how to access alternative treatments, which is bolstered by the active promotion and legitimization of alternative treatments.

Liu [16] systematically assessed the beneficial and harmful effects of herbal medicines in people with HIV infection and AIDS. Based on a Cochrane review and updated searches, the author identified the available evidence on herbal medicines compared with placebo or antiretroviral drugs in patients with HIV infection, HIV-related disease or AIDS. There were ten randomised controlled trials, involving 571 individuals with HIV infection or AIDS that met the inclusion criteria. Some herbal medicines, such as IGM-1 seem to be effective in symptom improvement, but generally no significant effect on antiviral or immunity enhancement among reviewed herbs was seen. These findings suggest beneficial effects from some of the tested herbs but results from larger studies are needed to support this evidence in the future.

A patient's level of knowledge about HIV disease, a belief that ART is effective and prolongs life, and a recognition that poor adherence may result in viral resistance and treatment failure all impact favourably upon a patient's ability to adhere [17]. Beliefs about the medications (including traditional) themselves also play a role in adherence. Patients who report low confidence in the efficacy of the medications and perceive minimal benefits resulting from ART are less likely to be adherent [18]. Lack of faith in the medications combined with a poor outlook for the future, often leaves little motivation for adherence to prescribed pharmacotherapy. So far, the impact of TCAM on the quality of care and patient outcome is not known in South Africa. This study will therefore look at patient characteristics and beliefs in TCAM, patterns of use and perceived benefits. It is estimated from other studies [5-7] that at least 30% of patients on ART will use any form of TCAM.

The aim of this cross-sectional study was to assess the use of Traditional, Complementary and Alternative Medicine (TCAM) for HIV patients prior to antiretroviral therapy in three public hospitals in KwaZulu-Natal, South Africa.

## Methods

### Design

This is a cross-sectional study of all treatment naïve patients recruited from all three public hospitals in Uthukela health district in KwaZulu-Natal from October 2007 to February 2008. All ART-naïve patients who were about to commence ART (18 years and above) and who consecutively attended the HIV clinics during the recruitment period were eligible for this study. The Uthukela Health District has a population of 553 671 and comprises five local authority areas. The District has one regional and two district hospitals, one private hospital, three primary health care facilities, 24 fixed clinics and 17 mobile clinics with 177 visiting points [19]. Initiation to ART is done at the three public hospitals. Some patients are referred to primary care clinics for ARV collection but return to the hospital for six monthly visits.

### Sampling procedure and recruitment

Systematic sampling was used by asking health care providers for referrals of ART-naïve patients (having a CD4 count below 200, eligible for ARV treatment but who had not commenced ARV treatment yet). Physicians from the three selected public clinics asked every consecutively visiting ART-naïve patient meeting the inclusion criteria of being 18 years or over (until at least 100 ART-naïve patients were found per clinic) if they would like to complete a confidential survey and interview concerning their health and social situation. This would include information from their medical records on details of their medical condition, laboratory tests and treatment. It was made clear to patients that their participation in this study was voluntary and a decision not to participate would not affect their medical care. If the potential participant indicated an interest in participating, the health care provider then referred them to an external HSRC research assistant. Before the survey was administered, individuals were given background information on the study. ART-naïve patients were then asked to sign and complete a consent form before the interview took place in a private area in or outside the clinic. The interviews were conducted by four trained external HSRC researchers (one or two per HIV clinic) in interview administration of the semi-structure interview schedule. Permission to access patient medical records was sought from both the patient and the health worker/manager. Questionnaires were anonymised, with no personal identifying information recorded on them. Recruitment took place over a period of four months, with 97.8% participation rate. Data were collected using an interviewer-administered semi-structured questionnaire. The questionnaire was translated into the major language spoken in the study area (Zulu) and verified by a second translator. Where inconsistencies were found, these were corrected. Pre-testing of the questionnaire was completed with five HIV-positive persons not involved in the study.

#### Ethical approval

The study protocol was approved by the Human Sciences Research Council ethics committee, the KwaZulu-Natal Department of Health, the Uthukela health district and the three superintendents of the three study hospitals.

### Outcome measures

Background information included socio-demographic characteristics, clinical history and health-related characteristics. Clinical data relating to date of HIV diagnosis, HIV risk factors, current CD4 cell count, opportunistic infections, HIV and non-HIV medications was obtained from the medical chart [20].

#### The Revised Sign and Symptom Checklist for Persons with HIV Disease

The SSC-HIVrev is a 72-item checklist of HIV/AIDS-specific physical and psychological symptoms, scored using the following scale: 0 = not checked (not present today), 1 = mild, 2 = moderate, 3= severe [21]. Analysis excluded the eight gynaecological problems, only relevant to women. Calculations included the total number of symptoms checked as present today (with a range of 0–64) and the total symptom intensity is a weighting of symptoms checked by the 1-to-3 rating of *mild, moderate, or severe *[21]. Validity and reliability of the instrument have previously been reported for a U.S. sample [21] and various African countries including South Africa [22]. Reliability estimates were calculated using Cronbach's alpha, which was .95 in this sample for the 64-item checklist. The total HIV symptom frequency showed a significantly non-normal distribution [*D*(612) = 0.21, *P *< .001] (Kolmogorov-Smirnow test).

#### Traditional, complementary and alternative medicine (TCAM) measure

Data were collected on prevalence, patterns and costs of TCAM use in conjunction with conventional drugs. A verbal explanation of TCAM was provided to each participant as follows: Traditional, complementary and alternative medicine has been defined broadly. It includes everything from herbal medications such as St. John's wort, African potato, cannabis to spiritual practices and prayer. It also includes things like Traditional Chinese Medicines, acupuncture, acupressure, chiropractic care, and massage therapy. Techniques such as meditation, visualization, and therapeutic touch would also be included. In addition, micronutrients (vitamins, minerals, and multivitamins) are included. Specific questions were asked about 18 different categories of TCAM, as follows: 1) Herbal therapies (e.g., Ginseng, Echinacea or St. John's Wort, *Hypoxis *plant (African potato); 2) Homeopathic therapies; 3) Traditional Chinese Medicine (including acupuncture, acupressure or traditional Chinese practitioners); 4) Supplements, nutraceuticals or Natural Health Products (e.g., N-acetyl-cysteine [NAC], melatonin, whey proteins, chondroitin); 5) Aromatherapy oils; 6) Chiropractic care; 7) Massage; 8) Naturopathy; 9) Reflexology; 10) Hypnosis; 11) Faith healing methods; 12) Imagery or visualization techniques; 13) Meditation; 14) Spiritual practices or prayer; 15) Therapeutic touch; 16) Micronutrients (vitamins, etc.); 17) Cannabis; 18) Exercise [23-25]. For each agent reported, patients also provided the specific name of the remedy, costs and reasons for use and physician awareness of use [26].

#### HIV knowledge and patient-provider variables

Participants' **HIV-related knowledge and orientation towards treatment information **were assessed with two indicators. Self-assessed knowledge about HIV/AIDS was measured with a single item, "How well-informed do you rate yourself to be about HIV disease and treatment relative to most people who are HIV positive?" Response options were 1 = much better informed than most to 5 = much less informed than most. Responses indicating that they perceived themselves to be "much better informed than most" and "somewhat better informed than most" were categorized as better informed (*vs*. those who viewed themselves as being "about as well informed," "somewhat less informed", or "much less informed") [6].

The **desired level of information involvement **was assessed with two items, e.g. "I want to take an active role in the medical management of my HIV infection and its complications." Response options were 1 = strongly agree to 4 strongly disagree. These items were combined into a 4-point scale in which higher scores indicate a stronger desire for information [6]. Cronbach alpha for this information desire index was .77 for this sample.

Participants' **degree of trust in medical providers **was measured with two items, e.g. "How much do you trust your doctor or clinic to offer you high-quality medical care?" Response options were from 1 = completely to 5 = not at all. Responses to these questions were reversed, combined, and placed on a scale ranging from 0 to 100, in which higher scores indicate higher trust [6]. Cronbach alpha for this trust in medical providers' index was .88 for this sample.

**Desired level of decision involvement reflected **the extent to which participants wanted to be involved in medical decision making, e.g., "It is better to trust a doctor or a nurse in charge of a medical procedure than to question what they are doing". Response options were 1 = strongly agree to 4 strongly disagree. These items were combined into a 4-point scale in which higher scores indicate a stronger disagreement (i.e., greater desire for involvement in decisions about medical care) [6]. Cronbach alpha for this desire for involvement in decisions index was .72 for this sample.

The **experience of discrimination in the health care system **was assessed with 3 items, e.g. "Has anyone in the health care system ever exhibited hostility or a lack of respect toward you?" Response options were "yes" or "no". If the participant answered "yes" to any of the three items, he or she was coded has having experienced discrimination [6]. Cronbach alpha for this health care provider discrimination index was .77 for this sample.

#### Internalized AIDS stigma

Items were adapted to assess internalized AIDS stigmas from a scale developed to measure AIDS related stigma beliefs in general South African populations. We selected four items from the AIDS-Related Stigma Scale [27] and reframed the wording to represent negative self-perceptions and self-abasement in relation to being a person living with HIV/AIDS. The items focused on self-blame (e.g., "I sometimes feel worthless because I am HIV positive.") and concealment of HIV status from others (e.g., "I hide my HIV status from others."). In this study, we examined responses to each of the four internalized stigma items as individual indicators of internalized AIDS stigma and we computed a scale by summing all items endorsed in the direction of greater internalized stigma. Items were responded to from 1 = strongly agree to 4 = strongly disagree. Strongly agree and agree were converted to "1" and strongly disagree and disagree to "0"; scale scores represent the sum total of endorsed items, range 0–4. Cronbach alpha for this stigma index was .82 for this sample.

#### Health Beliefs

Four health beliefs were drawn from the Health Belief Model (HBM) [28,29] and the revised HBM as described by Ronis [30]. All beliefs are measured on a 5-point agreement scale. *Perceived benefits *is a 2-item scale reflecting what patients perceive they would derive from their treatment adherence behaviours (e.g. prolonging life and decreasing chance of getting worse) to TCAM respectively (Cronbach alpha = .58). *Perceived barriers *is a 4-item scale reflecting what patients perceive they would have to overcome to adhere to TCAM. Perceived barriers were explored as non-monetary (e.g. stress of treatment, side effects of drugs, interrupted social activities) and monetary (e.g. expenditures on drugs, TCAM) (Cronbach alpha = .80). *Perceived severity *was the perceived seriousness of the disease and is measured in comparison to other illnesses (4 items) (Cronbach alpha = .78). [31].

### Statistical analysis

Prevalence data as well as predictors of use were evaluated for herb use and TCAM use. For subsequent analyses, participants were categorized as herb or TCAM users or nonusers, where users are those patients reporting the use of one or more herb or TCAM substances, depending on the specific analysis being conducted. Variables were inspected for skewness and those that domonstrated significant divergence from normality were transformed using *Log*_10 _[X+1] for regression analyses. Demographics, non-prescription medication use, disease characteristics (including CD4 count) were assessed in relation to TCAM use/nonuse through bivariate analyses. Estimated rates of TCAM use was calculated and bivariate associations between the independent variables and any use of TCAM for HIV in the past six months were investigated. Multivariate logistic regression was used to estimate the influence of each independent variable on the odds of using herbs and TCAM; included in the model were all variables significant at *P *< .05 in bivariate analysis. Collinearity diagnostics was conducted and all explanatory variables (socio-demographic and health variable factors) had tolerance values of .8 and more indicating no multicollinearity. The 95% confidence intervals for odds ratios and p values obtained by logistic regression were obtained using SPSS software, version 14.0 (Chicago, IL, United States). In multivariate regressions, adjustment was made for the hospital site, to account for TCAM variations and demographic differences across sites.

## Results

### Demographics

The sample included 618 Black African (almost all Zulu) HIV-positive patients (29.1% male and 70.9% female) prior to ART initiation, with an age range of 18 to 67 years (Median = 34.00 years; interquartile range = 12). Almost three-quarters of the sample were never married; only a quarter had completed secondary or post-secondary education and the largest proportion of patients (27%) belonged to the Zion Christian Church. The sample are further characterized by residing in rural areas (63.2%) and high unemployment (63%) (see Table 1).

**Table 1:** Baseline characteristics

**Variable**	**N = 618**	**%**
***Sex***		
Male	180	29.1
Female	438	70.9

***Age in years***		
18–29	167	27.0
30–39	269	43.5
40–49	121	19.6
50 and above	61	9.9

***Marital status***		
Never married	433	71.0
Currently married	73	12.0
Cohabitating	68	11.1
Divorced/separated	14	2.3
Widowed	22	3.6

***Highest education***		
None	42	6.8
Primary	447	72.7
Secondary	122	19.8
Post-secondary	4	.7

***Ethnicity***		
Zulu	612	99.0
Other	6	1.0

***Religious affiliation***		
African/traditional	58	9.4
Christian (Protestant churches)	88	14.2
Christian (Catholic)	40	6.5
Apostolic	57	9.2
Zion Christian Church	167	27.0
Other	102	16.5
No religion	106	17.2

***Residence***		
Rural village	285	46.3
Informal settlements (slums)	30	4.9
Urban/metropolitan areas	47	7.6
Township	150	24.4
Farm	104	16.9

***Employment situation***		
Housewife, home maker	86	14.1
Unemployed	383	63.0
Employed	117	19.2
Pensioner, student, disabled	22	3.6

***Main source of household income***		
Formal salary	182	29.4
Contribution by adult members	115	18.6
Government grant	138	22.3
Grants/donations by private welfare organizations	87	14.1
No income (other than social grant)	45	7.3
Other	51	8.3

### Health information

The majority (75.2%) of patients had received their HIV diagnosis within the last year and 93.9% of participants had a CD4 count below 200 cells/uL (*v*. Table 2), the criteria to be put on ART in the public health sector in South Africa. The participants reported that on the day of the interview, they were experiencing a median of 5.0 symptoms (interquartile range = 12.0) out of a possible 64. The top ten reported symptoms included concern over weight loss, dry mouth, headaches, memory loss, weakness, thirst, painful joints, chills (feeling very cold), chest pain and lack of appetite. Most of the participants (82.9%) felt that they were better informed than most about HIV disease and HIV treatment-related knowledge. Desired information involvement (56.7%) and degree of trust in the medical provider (62.1%) were both high, with medium levels of decision involvement (49.9%). Only a few (2.8%) reported to have experienced discrimination from the health care systems (2.8%) (*v*. Table 2).

**Table 2:** Health characteristics

**Variable**	**N = 618**	**%**
**Time since HIV diagnosis**		
≤ 1 year (2007/8)	454	75.2
1–2 years (2006)	61	10.1
> 2–3 years (2005)	30	5.0
> 3 years (2004–1995)	59	9.8

**CD4 count (cells/uL)**		
1–49	135	22.7
50–99	145	24.4
100–149	128	21.5
150–199	150	25.3
200 +	36	6.1

**Number of HIV symptoms **(range 0–55)	Median = 5.00	Interquartile range = 12.00

**HIV disease & HIV treatment related knowledge **(better informed than most)	509	82.9

**Information involvement score**		
4.0	345	56.7
3.0–3.9	255	41.9
< 3.0	9	1.5

**Degree of trust in medical provider score**		
100	380	62.1
75–99	207	33.8
0–74	25	4.1

**Discrimination by health care provider**	17	2.8

**Decision involvement score**		
4.0	281	46.8
3.0–3.9	300	49.9
< 3.0	20	3.3

More than half of the participants (56.9%) had visited a clinic three times or more in the past six months and 27.4% had been hospitalized. Very few respondents had home visits (2%), participated in a support group (1%) or had seen someone for counselling/support (6.5%).

TCAM was commonly used for HIV by study participants (317, 51.3%) and herbal therapies alone (183, 29.6%). The use of micronutrients (42.9%) was excluded from TCAM since mostly vitamins were provided by the health facility. Table 3 provides information about participants' use of major types of TCAM, the length of usage, costs in Rand per month and health care provider awareness about TCAM use. The range of using TCAM types ranged from 24 to 7 weeks (excluding cannabis, which was 38 weeks), with herbal therapies on average 12 weeks, faith healing 10 weeks and micronutrients (vitamins, etc.) 16.5 weeks. Herbal therapies were the most expensive, with one average 128 Rand (range 0–1000) per month, followed by cannabis (37 Rand per month), faith healing methods (24.4 Rand per month) and micronutrients 7.2 Rand per months (1 US$ = 7.60 Rand). Most participants (90%) indicated that their health care provider was not aware that they were taking herbal therapies for HIV (90%) or faith healing methods for HIV (81.6%), while most health care providers were aware that they were taking micronutrients (85.3%). TCAM use in females was particularly related to religious and spiritual practices. Women (42%) in this sample were much more likely to belong to charismatic churches than men (21%) and used faith healing methods (40% for women and 20% for men respectively) and spiritual practices or prayer (15% vs. 7%) more often than men (p < .001) (see Table 3).

**Table 3:** Use of TCAM (Traditional, complementary and alternative medicine) for HIV in the past six months (N = 618)

			**Use duration in weeks**	**Cost in Rand/month**	**Health care provider aware of use**
	**n**	**%**	**M (SD) [range]**	**M (SD) [range]**	**n (%)**

**Total TCAM use**^a^	317	51.3			

**1. Herbal therapies**	183	29.6			
1a. Herbal therapies	177	28.6	12.1 (9.0) [1–48]	128 (189) [0–1000]	18 (10.2)
1b. Cannabis	23	3.7	38.1 (37.5) [1–140]	37.3 (90.5) [0–400]	0

**2. Religious healing**	217	35.1			
2a. Spiritual practices or prayer	208	33.2	18.3 (10.1) [1–48]	0	69 (39.2)
2b. Faith healing methods	78	12.4	9.7 (9.5) [1–42]	24.4 (109.0) [0–500]	9 (18.4)

**3. Physical/body-mind therapy**	47	7.5			
3a. Exercise	29	4.6	12.6 (9.2) [1–28]	1.1 (3.3) [0–10]	8 (30.8)
3b. Massage	7	1.1	8.5 (10.9) [1–24]	10.0 (17.3) [0–30]	
3c. Therapeutic touch	6	1.0	10.0 (9.6) [2–24]	0	
3d. Meditation	5	0.8	24.0 [24-24]	0	

**4. Micronutrients (vitamins, minerals & multivitamin)**	266	42.9	16.5 (9.3) [1–48]	7.2 (69.1) [0–800]	227 (85.3)

**5. Over-the-counter drugs**	77	12.5	5.1 (6.4) [1–21]	4.3 (13.2) [1–99]	7 (10.9)

^a ^The use of micronutrients (42.9%) was excluded from TCAM since mostly vitamins were provided by the health facility

For each TCAM type participants were asked the reasons for its use. Herbal therapies were mainly used for pain relief (87.1%), immune supplementation (52.7%) and stress relief (50.9%); cannabis for stress relief (85.7%); spiritual practices or prayer for stress relief (77.6%), to improve overall wellbeing (74.7%) and pain relief (64.5%); faith healing methods for stress relief (80.4%); exercise to improve overall wellbeing (76.9%); micronutrients (vitamins, etc.) for pain relief (94%) and immune supplementation (81.3%) and over-the-counter drugs for pain relief (87.9%). Twenty-five (4.1%) of the participants believed that they could treat HIV solely with TCAM.

Bivariate analyses indicate that sex, age, educational level, having health insurance, CD4 count levels, time since HIV diagnosis and number of HIV symptoms were not associated with the use of herbs for HIV or HIV-related symptoms (*v*. Table 4). Age, having health insurance and time since HIV diagnosis were also not associated with TCAM use. Conversely, rural residence of patients, having no religious affiliation, not having money to meet needs, no income (other than social grant), family members not contributing to main source of household income, being on a disability grant (for chronic illness including AIDS) and fewer clinic visits were associated with the use of herbs for HIV or HIV-related symptoms. Female sex, secondary/postsecondary education, rural residence of patient, belonging to a charismatic church, not having money to meet needs, family members not contributing to main source of household income, having no income (other than social grant), being on a disability grant (for chronic illness including AIDS), fewer clinic visits in the past six months and more frequent HIV symptoms were associated with TCAM use. The use of over-the-counter drugs was significantly associated with herb and TCAM use (OR = 9.22, 5.14–16.53, *P *= .000 and OR= 38.33, 9.31–158.53, *P *= .000).

**Table 4:** Odds ratios (ORs) and 95% confidence intervals (95% CIs) for herb use and TCAM use, univariate and multivariate analyses

	**Univariate analyses**						**Multivariate analyses**			
	**Herb use**			**TCAM use**			**Herb use**		**TCAM use**	

	**%**	**OR (95% CI)**	**P**	**%**	**OR (95% CI)**	**P**	**OR (95% CI)**	**P**	**OR (95% CI)**	**P**

**Sex**										
Male (n = 180)	29.9	1.00 (reference)		42.9	1.00 (ref)				1.00 (ref)	
Female (n = 438)	29.3	0.97 (0.66–1.42)	.86	54.8	1.61 (1.13–2.30)	.008			1.23 (0.63–2.61)	.501

**Age in years**		1.00 (0.98–1.02)	.89		0.99 (0.97–1.01)	.25				

**Educational level**										
None (n = 42)	21.4	1.00 (ref)		45.2	1.00 (ref)				1.00 (ref)	
Primary (n = 446)	28.9	1.49 (0.69–3.21)	.305	48.4	1.14 (0.60–2.15)	.692			0.99 (0.33–3.03)	.996
Secondary/postsecondary (n = 126)	35.7	2.04 (0.90–4.64)	.090	65.1	2.26 (1.11–4.59)	.025			2.62 (0.77–8.87)	.122

**Residence**										
Urban (n = 227)	16.3	1.00 (ref)		40.5	1.00 (ref)		1.00 (ref)		1.00 (ref)	
Rural (n = 388)	37.6	3.00 (2.06–4.66)	.000	58.0	2.03 (1.45–2.83)	.000	1.53 (0.84–2.79)	.1.64	1.56 (0.84–1.99)	.157

**Religion**										
Christian (main stream) (n = 128)	21.1	1.00 (ref)		41.4	1.00 (ref)		1.00 (ref)		1.00 (ref)	
Charismatic (n = 230)	27.0	1.38 (0.83–2.31)	.220	53.5	1.63 (1.05–2.52)	.029	0.88 (0.44–1.79)	.726	1.23 (0.62–2.46)	.597
No religion (n = 106)	42.5	2.76 (1.56–4.90)	.001	52.8	1.59 (0.94–2.66)	.082	1.27 (0.53–3.03)	.588	1.15 (0.55–1.88)	.224
African/traditional (n = 58)	29.3	1.55 (0.77–3.15)	.224	32.8	0.69 (0.36–1.32)	.263	1.30 (0.50–3.25)	.594	0.35 (0.12–1.07)	.66

**Enough money to meet needs**										
Not at all (n = 294)	43.0	1.00 (ref)		63.2	1.00 (ref)		1.00 (ref)		1.00 (ref)	
A little (= 170)	19.4	0.32 (0.21–0.50)	.000	48.2	0.54 (0.37–0.80)	.002	0.53 (0.26–1.09)	.083	1.37 (0.76–2.45)	.293
Moderately/mostly/completely (n = 140)	14.0	0.22 (0.13–0.36)	.000	30.7	0.26 (0.17–0.39)	.000	0.69 (0.29–1.65)	.404	1.14 (0.60–2.17)	.686

**Main source of household income**										
Formal salary (n = 182)	29.1	1.00 (ref)		58.2	1.00 (ref)		1.00 (ref)		1.00 (ref)	
Contribution by family members (n = 115)	15.7	0.45 (0.25–0.82)	.009	25.2	0.24 (0.15–0.40)	.000	0.61 (0.29–1.31)	.207	0.32 (0.14–0.70)	.005
Government grant (n = 138)	39.9	1.61 (1.01–2.57)	.045	58.0	0.99 (0.63–1.55)	.961	1.62 (0.82–3.10)	.164	0.89 (0.43–1.47)	.745
Grants/donations by private welfare (n = 87)	21.8	0.68 (0.37–1.24)	.208	44.8	0.58 (0.35–0.98)	.040	0.50 (0.20–1.24)	.135	0.77 (0.19–0.84)	.578
No income (other than social grant) (n = 44)	54.5	2.92 (1.49–5.37)	.002	88.6	5.59 (2.11–14.85)	.001	0.24 (0.02–3.72)	.309	1.45 (0.21–9.88)	.706

**Being on a disability grant (chronic illness including AIDS) (n = 128)**										
No	16.1	1.00 (ref)		27.6	1.00 (ref)		1.00 (ref)		1.00 (ref)	
Yes	32.0	2.46 (1.54–3.93)	.000	72.7	6.98 (4.43–10.99)	.000	3.42 (1.65–7.11)	.001	7.85 (3.38–18.25)	.000

**Having a health insurance**										
Yes (n = 64)	28.1	1.00 (ref)		40.6	1.00 (ref)					
No (n = 548)	30.0	1.09 (0.61–1.94)	.76	52.8	1.64 (0.97–2.77)	.066				

**CD4 count**		1.00 (0.99–1.00)	.39		1.00 (1.00–1.01)	.049			1.57 (0.70–3.55)	.275

**Time since HIV diagnosis**										
≤ 1 year (n = 460)	28.9	1.00 (ref)		50.1	1.00 (ref)					
> 1 and more years (n = 151)	28.7	1.01 (0.67–1.52)	.95	52.0	0.93 (0.64–1.34)	.69				

**Number of clinic visits in past 6 months**										
Never or once (n = 116)	50.9	1.00 (ref)		69.0	1.00 (ref)		1.00 (ref)		1.00 (ref)	
Twice (n = 147)	21.1	0.26 (0.15–0.44)	.000	32.0	0.21	.000	0.64 (0.26–1.61)	.159	0.66 (0.23–1.89)	.443.
Trice or more (n = 348)	25.6	0.51 (0.33–0.80)	.000	53.2	0.51	.003	0.51 (0.23–1.12)	.047	0.97 (0.39–2.42)	951

**Number of HIV symptoms**		1.00 (0.99–1.02)	.64		1.03 (1.01–1.05)	.001			2.26 (1.28–3.99)	.005

Nagelkerke R Square							.19		.44	

Multivariate logistic regression with use of herbs for HIV as the dependent variable confirmed results from the univariate analysis for being on a disability grant and fewer clinic visits continued to be significantly associated with use of herbs (*v*. Table 4). The association between not enough money to meet needs, less income, religious affiliation and rural residence of the patient were no longer significant in the multivariate model. Multivariate logistic regression with TCAM use for HIV as the dependent variable confirmed results from the univariate analysis, namely that being on a disability grant, more frequent HIV symptoms, and family members not contributing to the main source of household income continued to be significantly associated with use of TCAM. The association between fewer clinic visits, CD4 count levels, not enough money to meet needs, rural residence of patient, sex, educational level and religious affiliation were no longer significant in the multivariate model (see Table 4).

Using a five-point scale (with 5 indicating very satisfied) patient ratings of their health status were significantly different between users (median = 4.00, interquartile range = 1; mean rank = 318.77) and non-users (median = 4.00, interquartile range = 1; mean rank = 292.59) of TCAM (Mann-Whitney U test: *Z *= -1.985, *P *= 0.47), but users of herbs (median = 4.00, interquartile range = 1; mean rank = 265.27) were significantly less satisfied with their health than non-users of herbs (median = 4.00, interquartile range = 2; mean rank = 322.22) (Mann-Whitney U test: *Z *= -3.897, *P *= .000). The majority of both TCAM and herb users and nonusers reported overall health as satisfied or very satisfied (median score for users and non-users = 4).

Self-rated HIV disease and treatment knowledge adjusted for educational level did not differ between herb and non-herb users (*OR *= 0.99, 0.84–1.18, *P *= .95), while TCAM users adjusted for educational level had significantly higher HIV disease and treatment knowledge than TCAM non-users (*OR *= 1.49, 1.25–1.78, *P *= .000).

In univariate analysis TCAM users scored significantly lower on information involvement, degree of trust in the medical provider and decision involvement than non TCAM users (*OR *= 0.39, 0.26–0.56, *P *= .000; *OR *= 0.96, 0.95–0.98, *P *= .000; and *OR *= 0.38, 0.27–0.56, *P *= .000, respectively), and in multivariate logistic regression only lower decision involvement (*OR *= 0.57, 0.35–0.93, *P *= .024) was associated with TCAM use.

Internalized AIDS stigma (range 1 to 4, 4 being the highest) was with median 3.00 (interquartile range = 1.00) high. Lower internalized AIDS stigma was associated with higher use of herbs (*Z *= -2.151, *P *= .031) and TCAM (*Z *= -2.692, *P *= .007).

#### Health beliefs

Perceived benefits from TCAM was generally rated average (*Median *= 2.50), and perceived barriers to TCAM (*Median *= 3.00), and perceived severity of HIV (*Median *= 2.75) high. (*v*. Table 5). Perceived barriers of TCAM use were positively associated with TCAM use (Mann-Whitney U test *Z *= -4.36, P = .000) while perceived benefits of TCAM use (*Z *= -1.26, *P *= .21) and perceived severity of HIV disease (*Z *= -1.02, *P *= .31) were not associated with TCAM use (see Table 5).

**Table 5:** Health beliefs towards TCAM HIV treatment

**Domain**	**Variable**	**Median**	**Interquartile range**
**Perceived benefits **(Median = 2.50, Interquartile range = 1.00)	Adherence to TCAM HIV treatment can prolong my life	4.00^a^	2
	Adherence to TCAM HIV treatment can decrease the chances of getting worse	4.00	1
**Perceived barriers **(Median = 3.00, Interquartile range = 1.50)	Dealing with TCAM HIV treatment side effects is stressful	3.00	2
	TCAM HIV treatment cause annoying side effects	3.00	2
	Taking TCAM HIV treatment interferes a great deal with normal activities	3.00	2
	Taking TCAM HIV treatment costs a lot	2.00	3

**Perceived severity **(Median = 2.75, Interquartile range = 0.75)	My current infection with HIV will lead to serious long-term health problems (reverse scored)	3.00	2
	I will become very sick as a result of my infection with HIV (reverse scored)	3.00	1
	Compared to other illnesses, HIV infection is not serious	3.00	2
	Whenever I get sick it seems to be serious (reverse scored)	2.00	2

^a ^rated from 1 to 5, 5 being the highest

## Discussion

Amongst 618 HIV treatment naïve patients from three public hospitals in KwaZulu-Natal, TCAM was commonly used for HIV in the past six months by study participants (317, 51.3%) and herbal therapies alone (183, 29.6%). The use of micronutrients (42.9%) was excluded from TCAM since mostly vitamins were provided by the health facility. This finding compares with other studies in Africa and elsewhere. Langlois-Klassen *et al*. [12] found that the majority of AIDS out-patients (N = 87, 63.5%) in western Uganda were using traditional herbal medicine after HIV diagnosis. This seems to be for any type of health problem, while our study investigated TCAM specifically for HIV treatment. Roughly half (49%) of the HIV participants in an Australian study had used TCAMs to manage their HIV/AIDS [32]. The use of micronutrients (43%) in this study was similar among HIV positive patients in Thailand (51%) [7] but lower than HIV out-patients in Canada (89%) [23].

Most participants in this study indicated that their health care provider was not aware that they were taking herbal therapies (90%) and faith healing methods for HIV (81.6%). Furler *et al*. [23] found among Canadian HIV outpatients that more than half (53%) did not report any TCAM use to their treating physician. Given the potential for adverse reactions and drug interactions related to TCAM use, health care providers' awareness of TCAM use is crucial to optimize patient care [23]. Health care providers should be informed about this to include inquiring about herbal and TCAM use for both HIV-related illnesses and other comorbid conditions as part of their history taking and clinical assessments [12].

Despite an apparent reluctance of the South African patients to inform the treating health care provider of TCAM use, the vast majority of patients in our study reported a high degree of satisfaction with the conventional medical treatment they were receiving. However, TCAM users as well as herb users alone were significantly less satisfied with their health care provider than TCAM as well as herb non-users, which is conform to a study by Sutherland and Verhoef [33]. This study found that TCAM users scored significantly lower on decision involvement than non TCAM users. The opposite was found among American TCAM users [6].

Herbal therapies were mainly used for pain relief, immune supplementation and stress relief, spiritual practices or prayer for stress relief, to improve overall wellbeing and pain relief, faith healing methods for stress relief, exercise to improve overall wellbeing, and micronutrients (vitamins, etc.) for pain relief and immune supplementation. Only a few (4.1%) of the participants believed that they could treat HIV solely with TCAM. Furler *et al*. [23] found among HIV-infected outpatients in Ontario, Canada, that the most common patient-cited reason for TCAM use was general wellbeing, followed by relaxation, pain, stress, spiritualism and healing; 9% believed that it was possible to treat HIV solely with the use of TCAM. Dhalla *et al*. [5] found among HIV patients in British Columbia that major reasons for CAM use were to improve energy level, to supplement dietary intake and to enhance immune response, and in a U.S. sample, Kirksey *et al*. [24] found that the most common treated conditions by TCAM were anxiety/fear, depression, pain and neuropathy. Among ethnically diverse patients in the U.S. Coleman *et al*. [34] found that prayer was used as a complementary health strategy for HIV-related anxiety, depression, fatigue, and nausea. In this South African study, cannabis was mainly used for stress relief (85.7%) and to a lesser extent for recreational purposes (relaxation) (23.5%) and pain relief (17.6%). Cannabis in South Africa is grown especially in the rural areas illegally and sold in different quantities depending on the users needs [35]. In rural South Africa, cannabis is also grown for personal usage. Fogarty *et al*. [36] also found that 44.3% of an Australian HIV patient sample reported mixed use of marijuana for therapeutic and recreational purposes. Further research might consider possible interactions between cannabinoids and antiretroviral treatments, potential use of oral THC and the difficulties faced by clinicians and PLWHA in discussing cannabis use in the current legal context.

Higher HIV symptom frequency was associated with more frequent TCAM use but not herbal therapy use. Reports from developed countries indicate an association between increased use of complementary therapies and higher degrees of suffering [e.g. [37]]. Langlois-Klassen *et al*. [12] also found that the majority of the AIDS patients in their study in western Uganda used traditional herbal medicine for fever and pain.

The use of over-the-counter drugs was found to be highly associated with herb and TCAM use in this study, which conforms to other studies [e.g. [23]].

This study found female HIV patients were also engaged more frequently in TCAM use than male patients, while herbal therapies were used to similar extents by both male and female HIV patients. Female patients, particularly those who belonged to charismatic churches, used greater amounts of faith healing and spiritual care practices than males in this sample. In Mozambique, Pfeiffer [38] found that poorer women with sick children preferred to use the prophets in the African Independent Churches (AIC) or charismatic churches instead of traditional healers as they didn't have to pay at the prophets. The popularity of the AICs in this context has been attributed to growing social inequalities which have resulted in heightened social tensions, violence and individual competition for jobs and social improvement, declining social cohesion and increased spousal conflict in poorer families [39]. With traditional healers catering mostly for men wishing to improve their luck and job prospects, many women have turned to the AICs. As well as finding traditional healers too expensive, many women worry that they themselves may be accused of witchcraft. The AICs have offered these women a new healing process which encourages mutual aid and support and avoidance of conflict. Other studies [e.g. [12]] found that female patients generally more frequently utilize traditional health medicine or TCAM [23,39] than male patients.

Herbal therapies were the most expensive, costing on average 128 Rand per patient per month. Other studies in South Africa even found higher costs of traditional herbal HIV medicines, 27–45 British pounds as reported by patients [13]. The reported costs for TCAM, at up to 100 US$ per month, represent a substantial proportion of the income of an average patient attending the clinic. This study found that the use of herbs for HIV was associated with being on a disability grant and fewer clinic visits of the patient. TCAM use for HIV was associated with being on a disability grant, "family members do not contribute to main source of household income", and number of HIV symptoms. Furler *et al*. [23] also found among HIV out-patients in Canada that TCAM users were more often receiving disability benefits than non TCAM users. The high costs of TCAM meant that users were not necessarily able to use them as much as they might have liked [*cf *also [32]]. One of the main types of TCAM use was that of prayer (no cost) and faith healing methods, which on average was lower in cost than herbal remedies. Studies amongst Black African HIV-positive patients in the UK have found that religion, as well as providing a spiritual coping mechanism for dealing with difficult life events, also provided practical support as a family might [40-42]. Studies have also found however that religious and spiritual beliefs may conflict with mainstream HIV treatment and care, particularly if patients believe that prayer alone may cure them [41,43].

Public health services including antiretroviral treatment are free in South Africa for primary health care and for hospital care a small user fee is collected. Despite relatively good access to conventional health care services, limited affordability of traditional health care including complementary care, and the current incurability of HIV/AIDS appear to restrict the use of TCAM. Likewise, limited affordability to access public health services may explain why AIDS patients in this study used TCAM. Patients must visit a treatment clinic at least six times in the year in which they start ART. The average cost per visit is R120, plus travel and waiting time [44].

In our analysis, we found no associations between TCAM and or herb use and years since HIV diagnosis and CD4 count. Most patients in this sample had a low CD4 cell count (below 200) and most had been diagnosed very recently with HIV. Other previous studies in HIV also failed to find a significant difference between users and nonusers of TCAM in terms of disease characteristics [e.g. [23,37]]. Other studies have, however, shown significant correlations between lower CD4 cell count [45] and time since diagnosis [39,46].

### Study limitations

The study findings may not be generalizable to HIV treatment naïve patients of public health facilities outside the Uthukela health district in KwaZulu-Natal, South Africa, to HIV patients below 18 years of age, and to HIV treatment naïve patients who did not access public health services. The sample in this study included 29.1% male and 70.9% female HIV-positive patients. Results found equal herb use between the sexes and significantly more TCAM use among female than male HIV-positive patients. This may indicate a selection bias towards female HIV-positive patients. However, other studies in South and Southern Africa have shown similar female to male ratios. From a random sample of 1072 adult pre-ART and ART patients across three different sites (public urban hospital, peri-urban non-governmental organisation (NGO) clinic site, and a rural NGO clinic) in Gauteng Province, South Africa, 21% were male and 79% female [44]. Muula et al. [47] found that in most Southern African countries, proportionally more females are on HIV antiretroviral treatment than men, even when the higher HIV infection prevalence in females is accounted for; the majority of the reports had female: male ratio in treatment exceeding 1.6. Further, this study was using hospital patients, therefore the study results cannot be generalized to the general popluation of persons with living with HIV/AIDS in the study area/South Africa. The cross-sectional study design did not permit an investigation of the cause-effect relationship between HIV/AIDS and use of herbs and TCAM. Recall bias of study participants cannot be excluded. Participants were often discouraged by health care providers to use traditional medicines and although participants were interviewed by external research assistants they may not have revealed the real extent of TCAM use.

## Conclusion

Traditional herbal therapies and TCAM are commonly used by HIV treatment naïve outpatients of public health facilities in South Africa. In some cases TCAM use may have interactions causing a decrease in ART effectiveness or even toxicity. It is not surprising therefore that the South African ART guidelines do not encourage the concomitant use of ARV and traditional medicine. Further pharmacokinetic and pharmacodynamic studies are needed to identify the potential risks, benefits, and interactions or non-interactions associated with concomitant ARV drug and herbal medicines, and the effects of TCAM use amongst patients on ART. Health care providers should routinely screen patients on TCAM use when initiating ART and also during follow-up and monitoring, keeping in mind that these patients may not fully disclose other therapies. Clinicians should endeavor to rule out ART interactions with TCAM that may result in therapeutic failure, side effects, toxicity and perhaps non-adherence. A more holistic approach to patient health care, with greater acceptance and acknowledgement that patients may use other sources of treatment will help patients to build trust with their HIV physician or nurse. An open dialogue between health care provider and patient about the importance of religious and spiritual practices in their lives should also be taken into account. Although there is a certain degree of co-operation between the government and faith-based organizations, results from this study highlight the need for further engagement between the two. Mapping both tangible and intangible religious health assets has proved useful in neighbouring countries in allowing gaps to be filled, resources to be channeled appropriately and interventions to be be designed [48]. An assets-based approach would therefore be of benefit in identifying, supporting and mobilizing religious health assets in this area. Patients should be involved more in the treatment decision-making process and provided with information on which aspects of TCAM can be safely incorporated into their medical regimen. Co-location of services including voluntary organizations offering spiritual and/or holistic health care advice may help to address patients' needs where health care providers lack the time or knowledge to adequately address these issues.

## Competing interests

The authors declare that they have no competing interests.

## Authors' contributions

KP and NF–DP conceptualized and designed the study, analysed and interpreted the data, drafted and revised the manuscript. SR participated in data collection, analysis and drafting of manuscript. HF participated in the design of the study and data analysis. All authors read and approved the final draft of the manuscript.

## Pre-publication history

The pre-publication history for this paper can be accessed here:


